# Serum Metabolomic Response of Myasthenia Gravis Patients to Chronic Prednisone Treatment

**DOI:** 10.1371/journal.pone.0102635

**Published:** 2014-07-17

**Authors:** Manjistha Sengupta, Amrita Cheema, Henry J. Kaminski, Linda L. Kusner, The Muscle Study Group

**Affiliations:** 1 Department of Neurology, George Washington University, Washington, DC, United States of America; 2 Department of Oncology, Lombardi Comprehensive Cancer Center, Georgetown University Medical Center, Washington, DC, United States of America; 3 Department of Pharmacology and Physiology, George Washington University, Washington, DC, United States of America; Istanbul University, Turkey

## Abstract

Prednisone is often used for the treatment of autoimmune and inflammatory diseases but they suffer from variable therapeutic responses and significant adverse effects. Serum biological markers that are modulated by chronic corticosteroid use have not been identified. Myasthenia gravis is an autoimmune neuromuscular disorder caused by antibodies directed against proteins present at the post-synaptic surface of neuromuscular junction resulting in weakness. The patients with myasthenia gravis are primarily treated with prednisone. We analyzed the metabolomic profile of serum collected from patients prior to and after 12 weeks of prednisone treatment during a clinical trial. Our aim was to identify metabolites that may be treatment responsive and be evaluated in future studies as potential biomarkers of efficacy or adverse effects. Ultra-performance liquid chromatography coupled with electro-spray quadrupole time of flight mass spectrometry was used to obtain comparative metabolomic and lipidomic profile. Untargeted metabolic profiling of serum showed a clear distinction between pre- and post- treatment groups. Chronic prednisone treatment caused upregulation of membrane associated glycerophospholipids: phosphatidylcholine, phosphatidylethanolamine, phosphatidylserine, 1, 2-diacyl-sn glycerol 3 phosphate and 1-Acyl-sn-glycero-3-phosphocholine. Arachidonic acid (AA) and AA derived pro-inflammatory eicosanoids such as 18-carboxy dinor leukotriene B4 and 15 hydroxyeicosatetraenoic acids were reduced. Perturbations in amino acid, carbohydrate, vitamin and lipid metabolism were observed. Chronic prednisone treatment caused increase in membrane associated glycerophospholipids, which may be associated with certain adverse effects. Decrease of AA and AA derived pro-inflammatory eicosanoids demonstrate that immunosuppression by corticosteroid is via suppression of pro-inflammatory pathways. The study identified metabolomic fingerprints that can now be validated as prednisone responsive biomarkers for the improvement in diagnostic accuracy and prediction of therapeutic outcome.

## Introduction

Corticosteroids are used for chronic treatment of numerous inflammatory and autoimmune disorders as well as diseases considered to have a significant inflammatory component [1]–[3]. Although effective for many conditions, their use is compromised by poor side effect profiles, which vary widely among patients [4]. To date, serum biological markers that are clearly modulated by chronic corticosteroid use have not been identified. Biomarkers are useful in medical practice as predictors of therapeutic effect or correlate to adverse effects. However, the discovery of these key determinants requires linkage to clinical trials to obtain samples. The effort is hampered by limited number of opportunities to obtain specimens, cost of collection, and lack of foresight by clinical trial organizers, thereby reducing the number of biological samples obtained from a homogenous population and treatment.

Myasthenia gravis (MG) is a chronic, autoimmune neuromuscular disorder caused by antibodies directed at proteins concentrated on the post-synaptic surface of the neuromuscular junction, primarily the nicotinic acetylcholine receptors. The primary treatment provided for patients with MG is prednisone administered for several months at high doses [5]. Typical treatment regimens result in at least 30 percent of patients having adverse effects due to dose and duration of administration [6], [7]. The sources of the variation in response to prednisone and susceptibility to complications among patients are not known and biological markers that are predictors of adverse effects or improvement have not been identified.

Variation in the response to pharmacologic agents have highlighted the necessity for individualizing drug therapy to select patients who are most likely to respond to treatment, to minimize the occurrence of adverse drug reactions, and to maximize the desired therapeutic effect. Metabolomics provides a snapshot of all the metabolites present at a given point of time, offers the opportunity for unbiased discovery of disease mechanisms, and identification of possible biomarkers of therapeutic responsiveness [8]. Metabolomic studies can assess therapeutic responsiveness from the perspective of a global alteration in metabolism, which is ultimately dependent on specific disease pathophysiology, individual subject variation (influenced by genetics and environmental factors), and drug mechanism. This technique has been used for determining drug response phenotype of several diseases [9].

Lu et al., used a serum metabolomic approach as a diagnostic measure to classify patients with various grades of MG [10] and identified a set of metabolites that could differentiate patients from healthy subjects. In this study, we used serum samples from fifteen patients for metabolomic analysis obtained during the course of a randomized clinical trial in which sampling was taken before treatment and after 20 mg of prednisone per day for treatment of generalized MG [11]. Our purpose was to explore the effect of prednisone treatment on metabolomic profile and identify treatment-responsive metabolites that could be translated to clinical applications. Ultra-performance liquid chromatography coupled with electro-spray quadrupole time of flight mass spectrometry (UPLC-ESI-QTOF-MS) was used to obtain comparative metabolomic and lipidomic profile of subject sera.

## Materials and Methods

### Ethics statement

The sera used in this investigation are de-identified samples from a clinical trial performed by the Muscle Study Group (NCT00683969). Internal Review Board approval was obtained by all investigators of the Muscle Study Group to obtain sera for investigative studies. The George Washington University Internal Review Board also approved the use of samples.

### Sample Characteristics

All samples were obtained during the course of a clinical trial performed by the Muscle Study Group (ClinicalTrials.gov identifier NCT00683969) [11] and drawn as part of routine phlebotomy to evaluate acetylcholine receptor antibody levels at study initiation and after 12 weeks of prednisone treatment. Subjects had been given no specific instructions regarding fasting or timing of the blood sampling. Serum was isolated and stored at −80°C. The subjects had mild to moderate MG with serum antibodies to the acetylcholine receptor antibodies. All patients at study entrance were naïve to immunosuppressive therapy for at least 3 months. For this study we used 15 serum samples of the patients obtained at trial initiation prior to treatment and then at 12 weeks of treatment with prednisone. Subjects were followed in a double blind fashion and subject to validated clinical outcome assessments including adverse effect assessment. Individual patient characteristics were not available to the present investigators because of the established informed consent agreement during the trial. Seventy-seven percent of patients improved and about twenty-five percent complained of adverse effects [11]. By clinical trial exclusion criteria, no subject had evidence of significant systemic illness at onset of the trial and at the end of 12 weeks of treatment the adverse effect reports did not indicate hepatic or renal dysregulation.

### Metabolite extraction

Sample processing and initial analysis was performed blinded to the treatment status. The serum samples were thawed from −80°C storage on ice. For metabolite extraction, 175 µL of 66% acetonitrile (in water) containing internal standards [10 µL of debrisoquine (1 mg/mL) and 50 µL of 4, nitro-benzoic acid (1 mg/mL)] were added to 25 µL of serum. The samples were incubated on ice for 15 minutes and centrifuged at 14,000 rpm at 4°C for 20 minutes. The supernatant was transferred to a fresh tube and dried under vacuum. The dried samples were re-suspended in 100 µL of solvent A (98% water and 2% acetonitrile) for UPLC-ESI-QTOF-MS analysis.

### UPLC-ESI-QTOF-MS based data acquisition

Each sample (5 µL) was injected onto a reverse-phase 50×2.1 mm BEH 1.7 µm C18 column using an Acquity UPLC system (Waters Corporation, USA). The gradient mobile phase comprised of water containing 0.1% formic acid solution (A) and acetonitrile containing 0.1% formic acid solution (B). Each sample was resolved for 10 min at a flow rate of 0.5 ml/min. This approach has been extensively used for metabolomic profiling of biofluids; UPLC gradients conditions and the mass spectrometry parameters have been described in details [12]–[14]. The UPLC gradient consisted of 100% A for 0.5 min then a ramp of curve 6 to 60% B from 0.5 min to 4.5 min, then a ramp of curve 6 to 100% B from 4.5 to 8.0 min, a hold at 100% B until 9.0 min, then a ramp of curve 6 to 100% A from 9.0 min to 9.2 min, followed by a hold at 100% A until 10 minutes. The column eluent was introduced directly into the mass spectrometer by electrospray. The column temperature was maintained at 40°C. Mass spectrometry was performed on a Quadrupole-time-of-flight mass spectrometer operating in either negative or positive electrospray ionization mode with a capillary voltage of 3.2 KV and a sampling cone voltage of 35 V. The desolvation gas flow was 800 L/h and the temperature was set to 350°C. The cone gas flow was 50 L/h, and the source temperature was 150°C. The data was acquired in the V mode with scan time of 0.3 seconds, and inter-scan delay at 0.08 seconds. Accurate mass was maintained by infusing sulfadimethoxine (311.0814 *m/z*) in 50% aqueous acetonitrile (250 pg/µL) at a rate of 30 µL/min via the lockspray interface every 10 seconds. Data were acquired in centroid mode from 50 to 850 *m/z* mass range for TOF-MS scanning, in duplicates (technical replicates) for each sample in positive and negative ionization mode and checked for chromatographic reproducibility. For all profiling experiments, the sample queue was staggered by interspersing samples of the two groups to eliminate bias. The coefficient of variance (CV) for serum samples was found to be <10%. The profiling experiment was repeated thrice to check experimental reproducibility.

For untargeted metabolomics profiling, the extraction buffer was spiked with a known concentration of internal standards (4, nitrobenzoic acid in negative mode and debrisoquine in positive mode) to account for variability in the extraction procedure and for inconsistencies during the MS acquisition. Relative quantitation was achieved for molecular ions using the UPLC-QTOF system by taking a ratio of normalized intensity of the respective study groups for the ions of interest [15]–[17]. In order to ensure data quality and reliability, a test mix of standard metabolites was injected at the beginning and at the end of the batch and the extracted ion chromatograms were evaluated for mass accuracy and sensitivity with respect to intensity for the given standards [18].

### Data Pre-processing

Centroided and integrated UPLC-TOFMS data were pre-processed using the XCMS [19] software and normalized to the ion intensity of respective internal standards (4, nitrobenzoic acid in the negative mode and debrisoquine in the positive mode). The resultant data containing mass/charge (m/z) value, retention time (rt) and peak intensity were subjected to multivariate data analyses between the pre- (T1) and post- (T2) treatment groups to delineate significantly altered metabolites.

### Statistical analysis

Prior to statistical analyses, the data were Pareto scaled which is the recommended method for LCMS data since both medium and small features in the data are important. We used two independent multivariate analysis methods as a means of “in silico” validation; Orthogonal Partial Least Squares Discriminant Analysis (OPLS-DA) models [20] and Random Forests methods [21]. The OPLS-DA models were run using SIMCA-P^+^ version 12 (Umetrics, Inc.) and the Random Forests methods were run using several R packages with specific use of the G plot library for heat map graphics within R. 2.11.0 [22]. Quantitative descriptors of model quality for the OPLS-DA models included R^2^ = 0.95 (explained variation of the binary outcome) and Q^2^ = .74 (cross-validation based predicted variation of the binary outcome) using two components. We used score plots to visualize the discriminating properties of the OPLS-DA models, and also S-plots for putative biomarker identification by visualization of the OPLS-DA loadings on the predictive score. Selection of features based on the OPLS-DA model used a p (corr) cut-off of 0.8, as reported previously [23], [24], which led to selection of the top 50 features. Further, we chose cross validation over permutation test since our data sets (number of samples in each group) were small. In our analyses, we employed 10-fold cross validation method, to determine the optimal number of components in our model. The features selected via OPLS-DA together with those selected as the top 50 discriminating features by the Random Forests methods were used for accurate mass based search. Top 50 metabolites that are differentially expressed in the two groups were selected and heat map was generated for both positive ionization and negative ionization data. We also performed a 2-tailed t-test on the entire data set and selected metabolites with p<0.05 for further evaluation. We used Graph Pad Prism 5 for depicting fold changes of selected metabolites as scatter plots.

### Metabolite identification

The metabolites were putatively identified by accurate mass based search using “MetaboSearch” (http://omics.georgetown.edu/MetaboSearch.html), a web-based software tool that enables simultaneous mass-based search against the four major databases, Human Metabolome DataBase (HMDB), Madison Metabolomics Consortium Database (MMCD), Metlin, and LIPID MAPS [25]. The output of the search provided an integrated table with m/z value of each feature, their name, formula, exact mass and fold change in expression. Based on this information, their putative identification was determined.

### Metabolomic Analysis

Kyoto Encyclopedia of Gene and Genome (KEGG) Pathway program (http://www.genome.jp/kegg/pathway.html) was used to identify the metabolic pathways that were deregulated in our study. We selected a few of the specific upstream or downstream components of each of the pathways and searched for their presence in our original data set by comparing their predicted mass values. This approach of “*in-silico*” validation was used to delineate the pathway involved.

### Validation

The metabolite identifications were confirmed by comparing the retention time under the same chromatographic conditions and by matching the fragmentation pattern of the parent ion from the biological sample to that of the standard metabolite using tandem mass spectrometry (UPLC-TOFMS/MS).

## Results

### Serum metabolomic analysis

**Figure 1:**
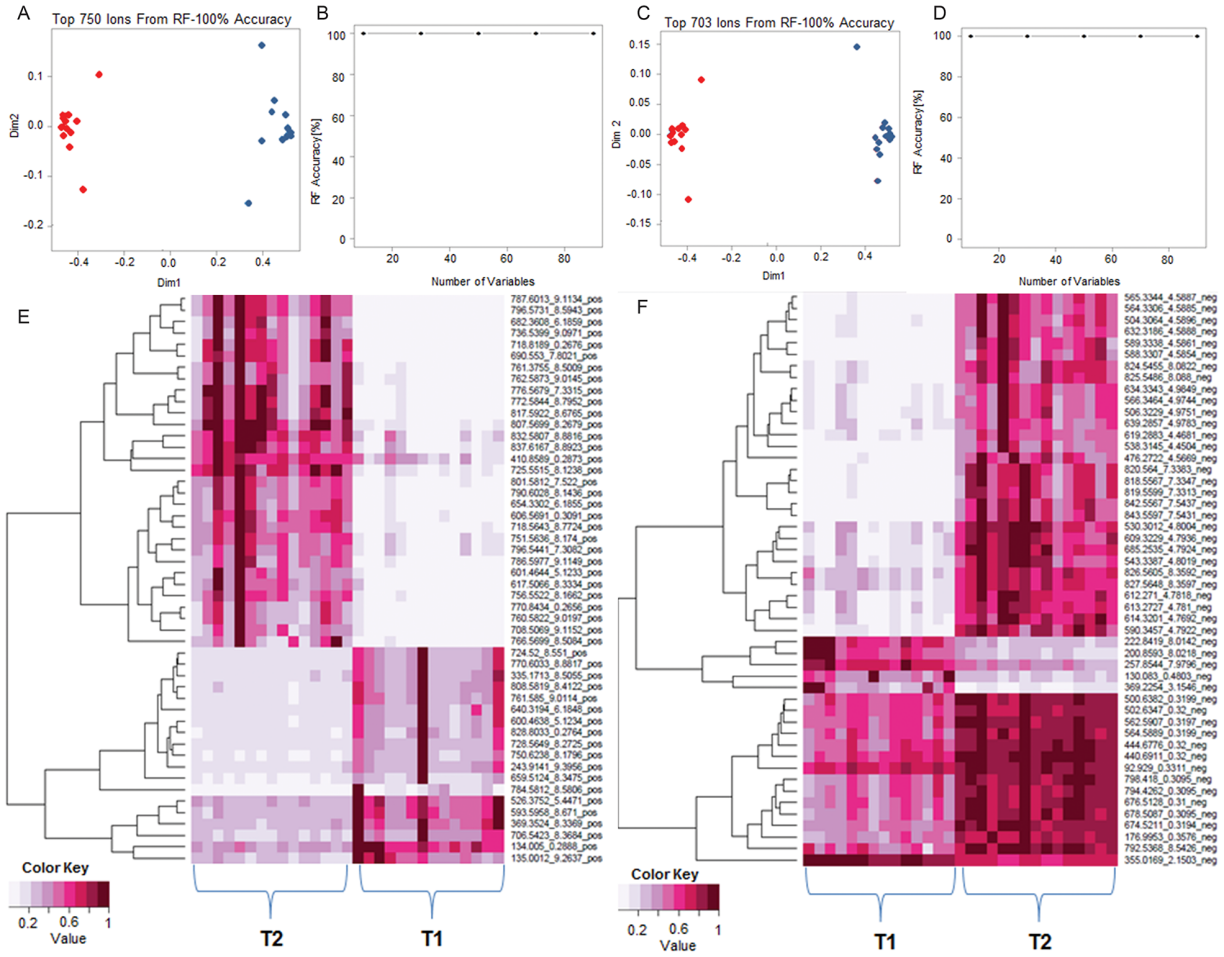
Prednisone treatment of MG patients shows clear segregation of metabolite patterns in positive and negative ionization mode. **A)** Two dimensional accuracy plot for 750 features interrogated in positive ionization mode using Random Forests (RF). It shows clear separation of the two groups T1 (before treatment in blue) and T2 (after treatment in red). X-axis denotes inter-class class separation and Y-axis shows intra-class variability **B)** Accuracy plot for RF in positive ionization mode shows 100% accuracy. **C)** Two dimensional accuracy plot for 703 features interrogated in negative ionization mode using Random Forests (RF). It shows clear separation of T1 and T2. **D)** Accuracy plot for RF in negative ionization shows 100% accuracy. **E)** Heat map visualization of the top 50 discriminating features between T1 and T2 in positive mode. **F)** The top 50 discriminating features between T1 and T2 in negative mode.

**Figure 2:**
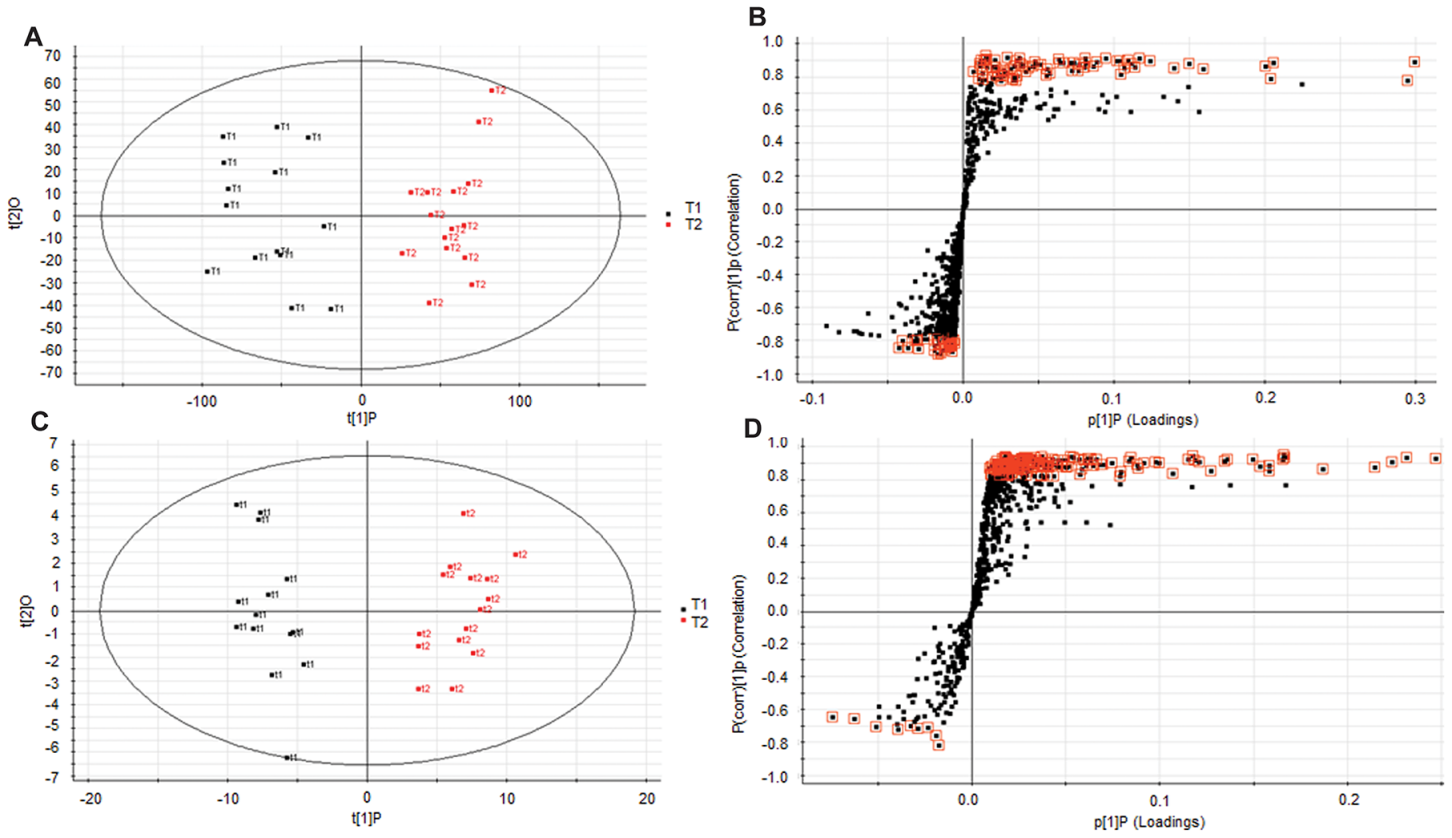
Orthogonal Partial Least Squares Discriminant Analysis (OPLS-DA) of determinants in positive and negative ionization mode. **A)** Scores plot obtained from OPLS-DA model of T1 and T2 in positive ionization mode shows inter-group separation **B)** OPLS-DA loadings S-plot comparing features from T1 and T2 in positive ionization mode. **C)** Score plot obtained from OPLS-DA model of T1 and T2 in negative ionization mode shows inter-group separation. **D)** OPLS-DA loadings S-plot comparing features from t1 and t2 in negative ionization mode. Each spot in S-plot corresponds to a feature with characteristic m/z ratio and retention time. The metabolites marked in red were treated as putative bio-markers for the therapy and characterized later.

Untargeted serum metabolomic profiling yielded 750 features in positive ionization mode and 703 features in negative ionization mode. Two dimensional accuracy plots for these features using Random Forests (RF) shows clear segregation of the two groups, T1 (before treatment) and T2 (after treatment) in both the ionization modes (Figures 1A and 1C). Accuracy plot shows 100% accuracy of class separation (Figures 1B and 1D). The dataset was filtered for metabolites with p-value ≤0.05. 50 metabolites that segregated baseline from post-treatment samples were analyzed using RF and heat maps were plotted (Figures 1E and 1F). The putative identities of each of these metabolites have been tabulated in the supplemental data (Tables S1 and S2). Orthogonal Partial Least Squares Discriminant Analysis (OPLS-DA) score plots were done using SIMCA-P^+^ (Figures 2A and 2C). The score plots resulted in an unambiguous inter-group separation. The loading plots (S-Plot) identified the metabolites with significant differences in the abundance in the two study groups (Figures 2B and 2D). The chemical formula calculations and putative identification of all these features were performed by an accurate mass based search by using the MetaboSearch software. Some of these metabolites were unambiguously identified using tandem MS by matching fragmentation pattern against standard compounds as described in the Validation section.

**Figure 3:**
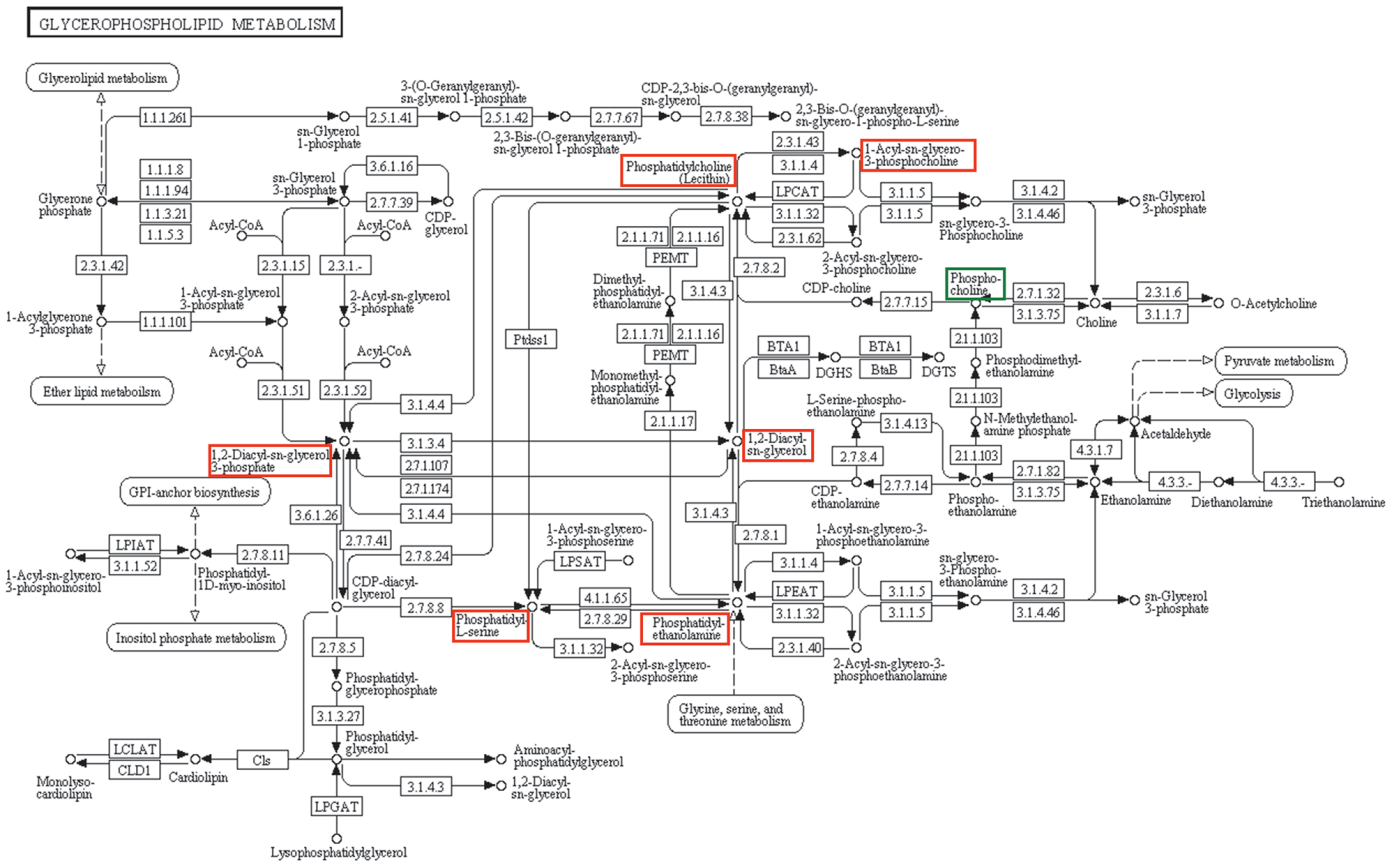
Prednisone treatment causes perturbation in Glycerophospholipid metabolism. The figure represents glycerophospholipid metabolism pathway from KEGG resource showing the metabolites that change significantly in MG patients after prednisone treatment. The metabolites marked in red shows increase in T2 (post-treatment) population and the ones marked in green shows decrease in abundance in T2. The figure is a screen shot from, http://www.genome.jp/kegg-bin/show_pathway?map=map00564&show_description=show.

Metabolomic profiling of serum samples showed statistically significant changes in glycerophospholipid metabolism (p≤0.05), in response to prednisone treatment. Within the glycerophospholipid metabolism pathway, phosphatidylcholine (PC) (4 to 11 fold; p = 6.89E-06), phosphatidylethanolamine (PE) (2 to 12 fold; p = 3.23E-08), phosphatidylserine (PS) (2 to 10 fold; p = 3.73E-08), 1,2-diacyl-sn glycerol 3 phosphate (phosphatidate, LPA) (2.12 fold; p = 3.27E-03), and 1-acyl-sn-glycero-3-phosphocholine (Lyso PC) (1.73 to 6.25 fold; p = 3.94E-3) showed increase in expression (Figures 3; Table 1). Phosphocholine demonstrated a decrease of 0.87 fold (p = 2.40E-02).

**Table 1:** Significantly altered metabolites of glycerophospholipid pathway.

Metabolite^1^	ID^2^	m/z^3^	RT^4^ (min)	Mode^5^	T2/T1^6^ Fold Change (+/−SEM)	p-value^7^
PC(18∶1/18∶1)	HMDB00593	786.5977	9.11	Pos	12.63 (1.98) ↑	7.4 E-09
PC(18∶3/P-8∶1)	HMDB08194	766.5699	8.5	Pos	8.72 (1.26) ↑	6.9 E-06
PC(16∶0/18∶3)	HMDB07975	756.5522	8.16	Pos	23.84 (6.90) ↑	7.5 E-10
PC(20∶0/14∶1)	HMDB08263	760.5822	9.01	Pos	20.09 (3.95) ↑	2.2 E-07
PC(20∶5/20∶2)	HMDB08506	832.5807	8.88	Pos	8.44 (1.77) ↑	2.8 E-10
PE(20∶1/18∶1)	HMDB09257	772.5844	8.79	Pos	17.25 (2.87) ↑	7.2 E-09
PE(20∶1/0∶0)	HMDB11512	506.3229	4.97	Neg	11.68 (2.78) ↑	3.5 E-10
PE(P-8∶1/18∶2)	HMDB11409	724.5314	8.71	Neg	3.33 (0.33) ↑	3.2 E-08
PE(20∶4)/P-8∶1)	HMDB09413	748.5297	8.61	Neg	5.26 (0.57) ↑	8.5 E-09
PE(18∶2/20∶4)	HMDB09103	762.5131	8.26	Neg	6.40 (0.59) ↑	1.8 E-08
PS(O-0∶0/17∶1)		790.6028	8.14	Pos	20.27 (5.53) ↑	7.2 E-10
PS(P- 8∶0/20∶4)		796.5441	7.3	Pos	19.95 (6.78) ↑	3.7 E-08
PS(19∶0/0∶0)		538.3145	4.45	Neg	38.97 (11.24) ↑	2.4 E-10
PS(17∶2/22∶2)		824.5455	8.08	Neg	12.70 (3.29) ↑	2.1 E-12
PS(18∶3/21∶0)		826.5605	8.35	Neg	4.76 (1.64) ↑	3.0 E-12
LysoPC(15∶0)	HMDB10381	480.3086	4.79	Neg	15.74 (4.81) ↑	9.3 E-12
LysoPC(20∶3)	C04230	546.3529	4.78	Pos	9.51 (6.63) ↑	5.2 E-07
LysoPC(18∶1)	C04230	522.3543	4.97	Pos	2.12 (0.39) ↑	3.9 E-03
LPA (0∶0/16∶0)	C00416	409.2348	5.21	Neg	11.41 (5.14) ↑	3.3 E-03
Phospho choline	C00588	184.0742	7.58	Pos	0.89 (0.05) ↓	2.40E-02

1 Glycerophospholipids (Phosphatidylcholine: PC, Phosphatidylethanolamine: PE, Phosphatidylserine: PS, lyso PC: 1-Acyl-sn-glycero-3-phosphocholine; LPA: 1,2-Diacyl-sn glycerol 3 phosphate or Phosphatidate).

2 Metabolite ID obtained from Human Metabolome Data Base and KEGG.

3 mass/charge value.

4 retention time.

5 Ionization modes.

6 Fold changes between post-treatment (T2) and pre-treatment (T1) groups with +/− SEM.

7 The p-value as determined by student’s t-test.

**Figure 4:**
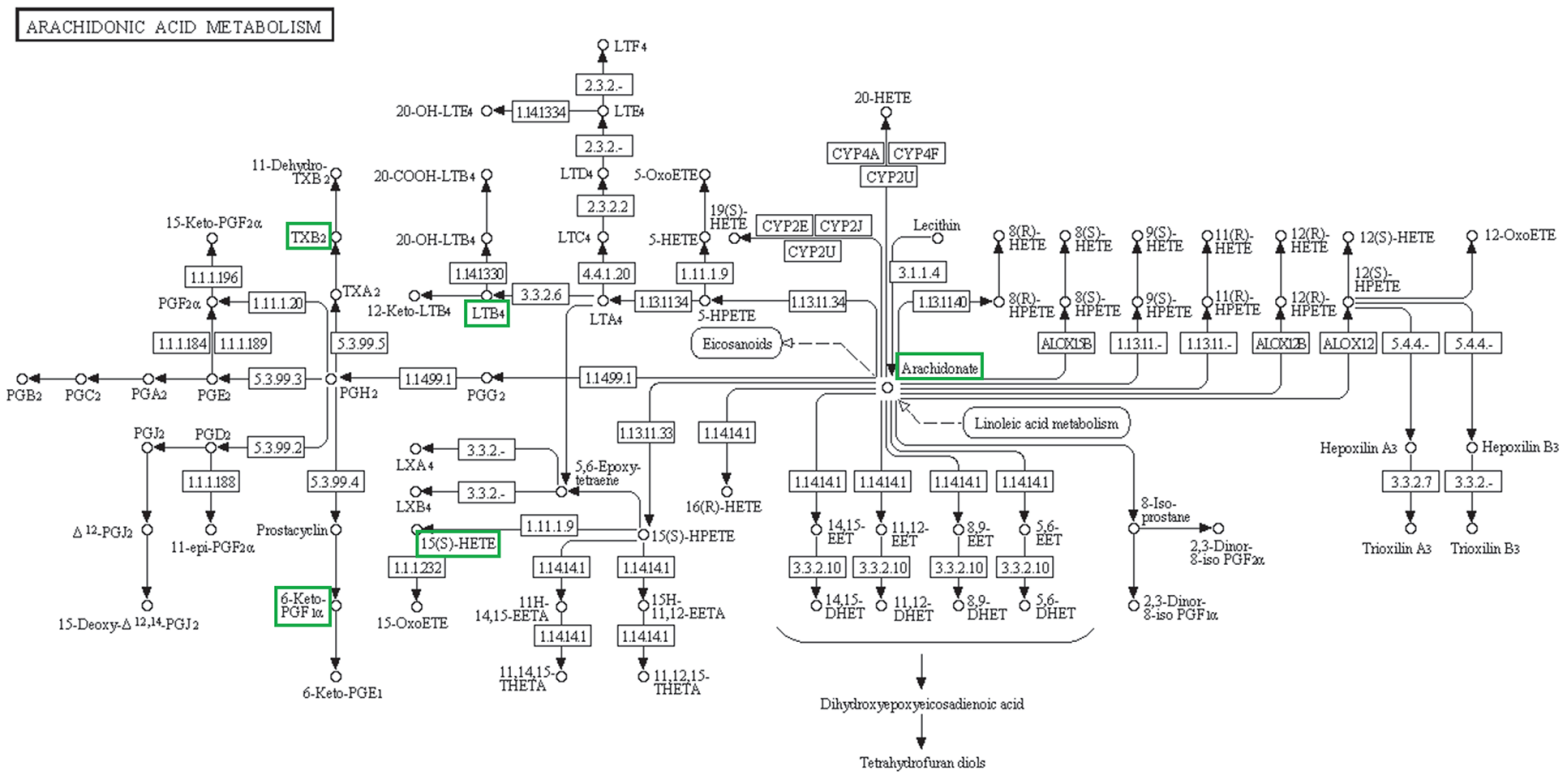
Downregulation of Arachidonic Acid (AA) and pro-inflammatory pathways. The figure represents arachidonic acid metabolism pathway from KEGG resource showing the metabolites that are downregulated in MG patients in response to prednisone treatment. The figure is a screen shot from, http://www.genome.jp/kegg-bin/show_pathway?map=map00590&show_description=show.

**Figure 5:**
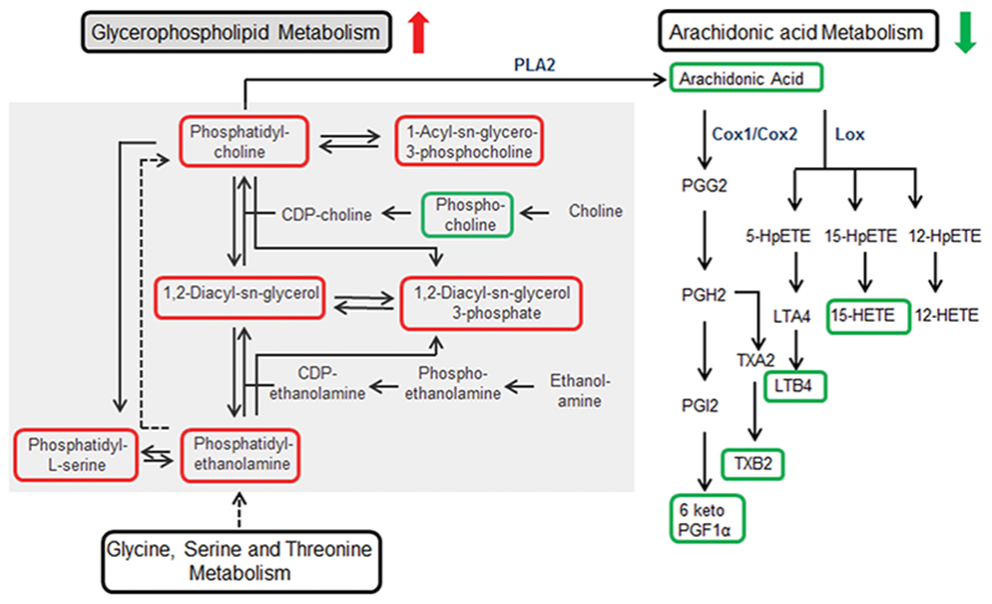
Metabolic changes in the serum samples of MG patients in response to prednisone treatment. Schematic outline summarizing the significant changes observed in the metabolic pathways. The upregulated metabolites identified in the serum samples have been marked in red and the downregulated metabolites have been marked in green. Membrane glycerophospholipids are upregulated in response to prednisone treatment. Arachidonic Acid (AA) metabolism via cyclooxygenase (COX) and lipoxygenase (LOX) pathway is downregulated causing reduction of pro-inflammatory metabolites. PG, Prostaglandin, TX, Thromboxane; LT, Leukotriene; HETE, Hydroxy-eicosatetraenoic acid; HpETE, Hydroperoxy-eicosatetraenoic acid; PLA, Phospholipase A.

Arachidonic acid metabolism and related anti-inflammatory pathway was found to be downregulated by prednisone treatment (Figures 4 and 5, Table 2). Downregulation of AA (0.42 fold; p = 4.46E-04), 18-carboxy dinor leukotriene B4 (0.77 fold; p = 2.5E-04) and 15 hydroxyeicosatetraenoic acids (15HETE) (0.34 fold; p = 1.01E-03) was observed. We have identified a metabolite with m/z value 369.2254 in negative ionization mode which corresponds with thromboxane B2 and 6-Keto prostaglandin F1alpha. Determining the exact identity of the metabolite was beyond the scope of the study but both the putative metabolites are present downstream of cox1/cox2 pathway of prostaglandin G2 metabolism. Decrease in the expression of the above mentioned metabolite by 0.29 fold (p = 7.84E-05) was observed.

**Table 2:** Significantly altered metabolites in pre and post treatment groups.

Pathway^1^	Metabolite^2^	ID^3^	m/z^4^	RT^5^ (min)	Mode^6^	T2/T1^7^ Fold Change (+/−SEM)	p-value^8^
Arachidonic acid metabolism	Arachidonic Acid	C00219	303.2326	5.97	Neg	0.55 (0.09) ↓	4.5 E-04
	15-HETE		319.2273	4.82	Neg	0.42 (0.06) ↓	1.0 E-03
	18-carboxy dinor Leukotriene B4		339.1811	6.57	Pos	0.80 (0.04) ↓	2.5 E-04
	Thromboxane B2/6-keto PGF1alpha	C05963	369.2254	3.15	Neg	0.33 (0.04) ↓	7.8 E-05
Amino acid metabolism	L-Phenylalanine	C00079	164.0718	0.66	Neg	0.70 (0.04) ↓	7.6 E-04
	O-Acetylserine	C00979	146.0462	0.33	Neg	0.79 (0.16) ↓	1.4 E-02
	N2-Acetyl-L-ornithine	C00437	175.1074	0.3	Pos	0.83 (0.12) ↓	1.2 E-02
Citric Acid Cycle	Aconitate Ion		171.9994	9.51	Pos	0.75 (0.04) ↓	6.4 E-06
	3-Epihydroxy-2′-deoxymugineic acid		321.1301	2.19	Pos	0.73 (0.06) ↓	3.8 E-03
Vitamins	3-Deoxyvitamin D3		369.3524	8.33	Pos	0.53 (0.04) ↓	1.9 E-05
	Vitamin E		531.4064	8.2	Pos	1.73 (0.22) ↑	1.4 E-02
Others	Hydroxy-Spheroidenone	C15905	601.4644	5.12	Pos	6.90 (0.85) ↑	7.2 E-08

1 Metabolic pathway involved.

2 Metabolites.

3 Metabolite ID obtained from KEGG.

4 mass/charge value.

5 Retention Time.

6 Ionization modes.

7 Fold changes between post-treatment (T2) and pre-treatment (T1) groups with +/−SEM.

8 The p-value as determined by student’s t-test.

Other metabolites that showed statistically significant alterations in expression were L-phenylalanine (0.66 fold; p = 7.63E-04), O-acetylserine (0.55 fold, p = 1.45E-02) and N2-acetyl-L-ornithine (0.72 fold; p = 1.24E-02); involved in amino acid metabolism. Citric acid cycle demonstrated perturbation, as noted by decrease in aconitate ion (0.731; p = 6.43E-6) and 3-epihydroxy-2′-deoxymugineic acid (0.67 fold; p = 3.78E-03). Vitamin E succinate (1.41 fold; p = 1.39E-02) showed an increase and 3-deoxyvitamin D3 (0.49 fold; p = 1.86E-05) showed a decrease in abundance in the post-treatment group. Hydroxyspheroidenone, involved in carotenoid biosynthesis, was increased by 6.27 fold (p = 7.25E-08) (Table 2).

To further assess variation in fold changes of the sample sets, we produced scatter plots depicting the mean fold changes (± standard error of the mean) (Figure S1). For each of the 32 metabolites, the 15 sets demonstrated the same increase or decrease in fold change trend in for all 32 metabolites illustrated.

### Validation

Validation of PC, PE and AA was done by UPLC-TOFMS/MS by comparing the fragmentation pattern of the standards with the ones present in the samples. We matched the parent and the daughter ions and confirmed the presence of PC (m/z 184.07, 185.07, 786.59, 787.60, 788.60), PE (m/z 184.07, 185.07, 772.58) and AA (m/z 259.24, 303.23) in our samples. See Figures S2, S3, and S4.

## Discussion

Untargeted metabolic profiling of serum obtained from patients before and after 12 weeks of prednisone treatment demonstrated an unambiguous metabolic signature of chronic glucocorticoid treatment. Despite expected human variation on metabolism [26], there was a uniform and significant effect across subjects with treatment as determined by several methods of analysis. Independent multivariate statistical approaches clearly discriminated pre- and post-treatment samples as did the Random Forests method with complete separation of the groups with 100% accuracy with respect to the discriminating metabolites. Heat maps of the top 50 differentially expressed metabolites also confirmed the clear distinction between the pre- and post-treatment groups. Orthogonal Partial Least Squares Discriminant Analysis (OPLS-DA) was performed. The OPLS-DA score plots clearly showed inter-group segregation of samples from subjects who were prednisone-naïve and after 12 weeks of prednisone therapy. Assessment of fold changes identified metabolites with a significant fold change (p<0.05) with prednisone treatment demonstrated overall consistency with expected biological variability across subjects; however, outliers existed with significantly greater fold changes (Figure S1). Taken together the observations support a uniform metabolomic signature for chronic prednisone treatment.

Detailed analysis of the differentially regulated metabolites revealed a significant change in glycerophospholipid metabolism. Out of the top 50 discriminating features identified by RF and represented in the heat maps, the majority were glycerophospholipids (Table 1) signifying a strong impact of prednisone treatment on glycerophospholipid metabolism. Corticosteroid stimulated *de novo* synthesis of PC was observed in developing lung and was associated with an increase in choline-phosphate cytidylyltransferase activity [27]. Metabolomic analyses of bronchoalveolar lavage fluid from dexamethasone treated, experimentally induced asthma mouse model identified a similar increase in PC level associated with a decrease in choline level [28]. We found an increase in PC level and a decrease in intermediate phosphocholine level (Figures 5). PC is a critical component of the cellular membrane structure and is involved in lipid cell signaling. The synthesis of PC can occur through two pathways; CDP-choline pathway and PE methylation pathway [29]. The free choline that is incorporated into PC is recycled back to the pool through hydrolysis by the enzyme phospholipase D [30]. There is an overall increase in PC, PE, PS and the intermediate DAG (Figure 5) signifying that the entire PE-methylation pathway is up regulated in response to prednisone treatment.

Apart from the increase in glycerophospholipid synthesis, a significant decrease in AA and AA acid mediated pro-inflammatory metabolites (Figure 5) was observed. Biologically active lipids derived from AA have crucial roles in inflammation. Enzymatic oxidation of AA is mediated by cyclooxygenase (COX), lipoxygenase (LOX) and P450 epoxygenase pathways generating eicosanoids. COX enzymes metabolize AA to pro-inflammatory prostaglandins (PGs) and thromboxanes (TXs). LOX mediated pathway converts AA to leukotrienes (LTs), hydroxyeicosatetraenoic acids (HETEs) and lipoxins. Cytochrome P450 metabolizes AA to cell signaling molecules such as epoxyeicosatrienoic acids, HETEs and hydroperoxyeicosatetraenoic acids [31]. We found a downregulation of AA and downstream eicosanoids of COX and LOX pathways (Figure 5). Glucocorticoid inhibits prostaglandin synthesis by inhibiting translation of PG synthase enzyme [32]. AA release by phospholipase A2 (PLA2) is suppressed by glucocorticoids causing downregulation of prostaglandin and leukotriene synthesis [32]. The results are in accordance with the previous observations that immunosuppression by corticosteroid is via suppression of these pro-inflammatory pathways. These alterations were identified by serum metabolite analysis and have the potential for being monitored to assess prednisone associated alterations. Vitamin E succinate, which has anti-inflammatory properties, was increased [33]. 3-Deoxyvitamin D3, an anti-inflammatory metabolite, was reduced [34].

One action of glucocorticoids is to subvert metabolic pathways away from pro-inflammatory AA synthesis, towards anti-inflammatory endocannabinoid synthesis pathway [35]. Though there was an increase in phospholipids in response to prednisone treatment, a decrease in AA and AA derived eicosanoids was found. Endocannabinoids are local lipid messengers that induce immune- and neuro-protection through the activation of G-protein coupled cannabinoid receptors CB1 and CB2 [36]. Endocannabinoid pathway metabolites were not identified in the metabolomic screening, but given their rapid and transient production the present analysis cannot rule out their modulation by chronic steroid treatment.

Corticosteroids induce muscle wasting through several mechanisms including regulation of transcription, insulin sensitivity and calcium concentrations [37]. Expected increase in proteogenic amino acids were not observed in our study. However, reduction in serum levels of L-phenylalanine, O-acetylserine and N2-acetyl-L-ornithine was found indicating an imbalance in protein synthesis and degradation [38].

A decrease in aconitate ion and 3-epihydroxy-2′-deoxymugineic acid shows deregulation of energy metabolism. Although perturbation in citric acid cycle was noted, we expected a greater number of metabolic pathways to be effected by prednisone treatment. The inability of the metabolomic data bases to identify every metabolite was a hindrance to the study. A number of metabolites could not be identified by the data base search even though they show a significant differential expression (Tables S1 and S2). Similarly, presence of certain metabolites in the samples was unexpected. For example, a 6 fold increase in hydroxyspheroidenone, involved in carotenoid biosynthesis was found that is not known to be related to steroid treatment or myasthenia gravis.

The metabolomic profile reflects an individual’s immediate physiological status. Our study demonstrates the potential of metabolomics as a mean to discriminate and differentiate between treated and untreated groups. The patients as a whole improved due to the treatment. From our study we can clearly see a trend of suppression of anti-inflammatory pathways by prednisone treatment and an increase in membrane phospholipid synthesis (Figure 5). Altered PC metabolism has been suggested in asthma with increase in level of PC and decrease in level of lyso-PC [39] and Crohn’s disease where phospholipids were increased [40]. Differences in free choline and PC level in serum have been associated with early markers of risk of having cardiovascular disease (CVD) [41]. Our results suggest AA could be used to assess treatment response and we hypothesize that PC levels may correlated to tissue complication of steroid treatment.

No previous investigation has evaluated metabolomic modifications among humans with an autoimmune disorder treatment with chronic prednisone administration. Despite the powerful effects on human physiology of prednisone, we identified metabolic alterations to be limited primarily to glycerophospholipid and arachidonic acid pathway alterations. Our study is a pilot study and a proof of principle. Further studies are warranted for mechanistic study of drug-mediated effect and molecular targets.

## Supporting Information

Figure S1 **Scatter plot representation of the fold changes in selected metabolites in 15 patient samples.** SEM added. PC: phosphatidylcholine; PE: phosphatidylethanolamine; PS: phosphatidylserine; LysoPC: lyso phosphatidylcholine; LPA: phosphatidate; Ph Choline: phospho choline; AA: arachidonic Acid; 15-HETE: hydroxyeicosatetraenoic acids; 18 COOH-LTB: 18-carboxy dinor Leukotriene B4; TXB2: Thromboxane B2/6-keto PGF1alpha; L-Phe: L-Phenylalanine; OAS: O-Acetylserine; N-Ac-Or: N2-Acetyl-L-ornithine; EpiHDMA: 3-Epihydroxy-2′-deoxymugineic acid; Vit D3∶3-Deoxyvitamin D3; Vit E Succ: Vitamin E Succinate; OH-Sp: Hydroxy-Spheroidenone.
(TIF)

Figure S2 **Validation of Phosphatidyl choline in the sample by MS/MS.** Candidate marker was validated by UPLC-TOF-MS/MS. Q1 to Q3 transition of Phosphatidyl choline was compared with the ones in the sample (m/z 184.07, 185.07, 786.59, 787.60, 788.60).
(TIF)

Figure S3 **Validation of Phosphatidyl ethanolamine in the sample by MS/MS.** Candidate marker was validated by UPLC-TOF-MS/MS. Q1 to Q3 transition of Phosphatidyl ethanolamine was compared with the ones in the sample (m/z 184.07, 185.07, 772.58).
(TIF)

Figure S4 **Validation of Arachidonic acid in the sample by MS/MS Validation of metabolites in the sample by MS/MS.** Candidate marker was validated by UPLC-TOF-MS/MS. Q1 to Q3 transition of Arachidonic acid was compared with the ones in the sample (m/z 259.24, 303.23).
(TIF)

Table S1 **Random Forests top 50 metabolite putative identity lists in positive ionization mode.**
(DOCX)

Table S2 **Random Forests top 50 metabolite putative identity lists in negative ionization mode.**
(DOCX)
