# Supplementary figures and images for: Serum Metabolomic Response of Myasthenia Gravis Patients to Chronic Prednisone Treatment
Source: PLoS One. 2014 Jul 17;9(7):e102635. doi: 10.1371/journal.pone.0102635 (PMC4102553; doi:10.1371/journal.pone.0102635)

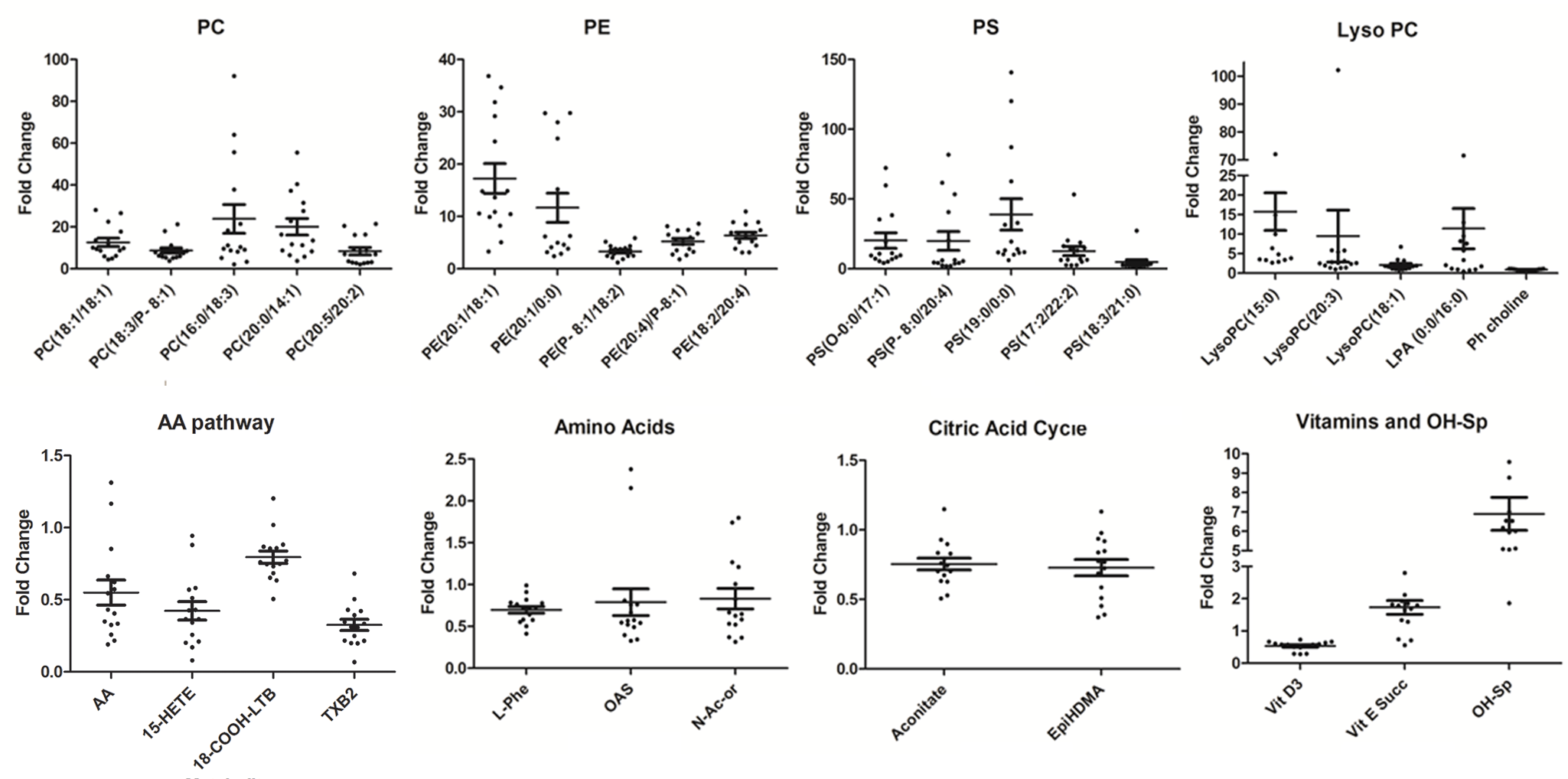

Supplement: Figure S1 — Scatter plot representation of the fold changes in selected metabolites in 15 patient samples. SEM added. PC: phosphatidylcholine; PE: phosphatidylethanolamine; PS: phosphatidylserine; LysoPC: lyso phosphatidylcholine; LPA: phosphatidate; Ph Choline: phospho choline; AA: arachidonic Acid; 15-HETE: hydroxyeicosatetraenoic acids; 18 COOH-LTB: 18-carboxy dinor Leukotriene B4; TXB2: Thromboxane B2/6-keto PGF1alpha; L-Phe: L-Phenylalanine; OAS: O-Acetylserine; N-Ac-Or: N2-Acetyl-L-ornithine; EpiHDMA: 3-Epihydroxy-2′-deoxymugineic acid; Vit D3∶3-Deoxyvitamin D3; Vit E Succ: Vitamin E Succinate; OH-Sp: Hydroxy-Spheroidenone. (TIF) [file pone.0102635.s001.tif]

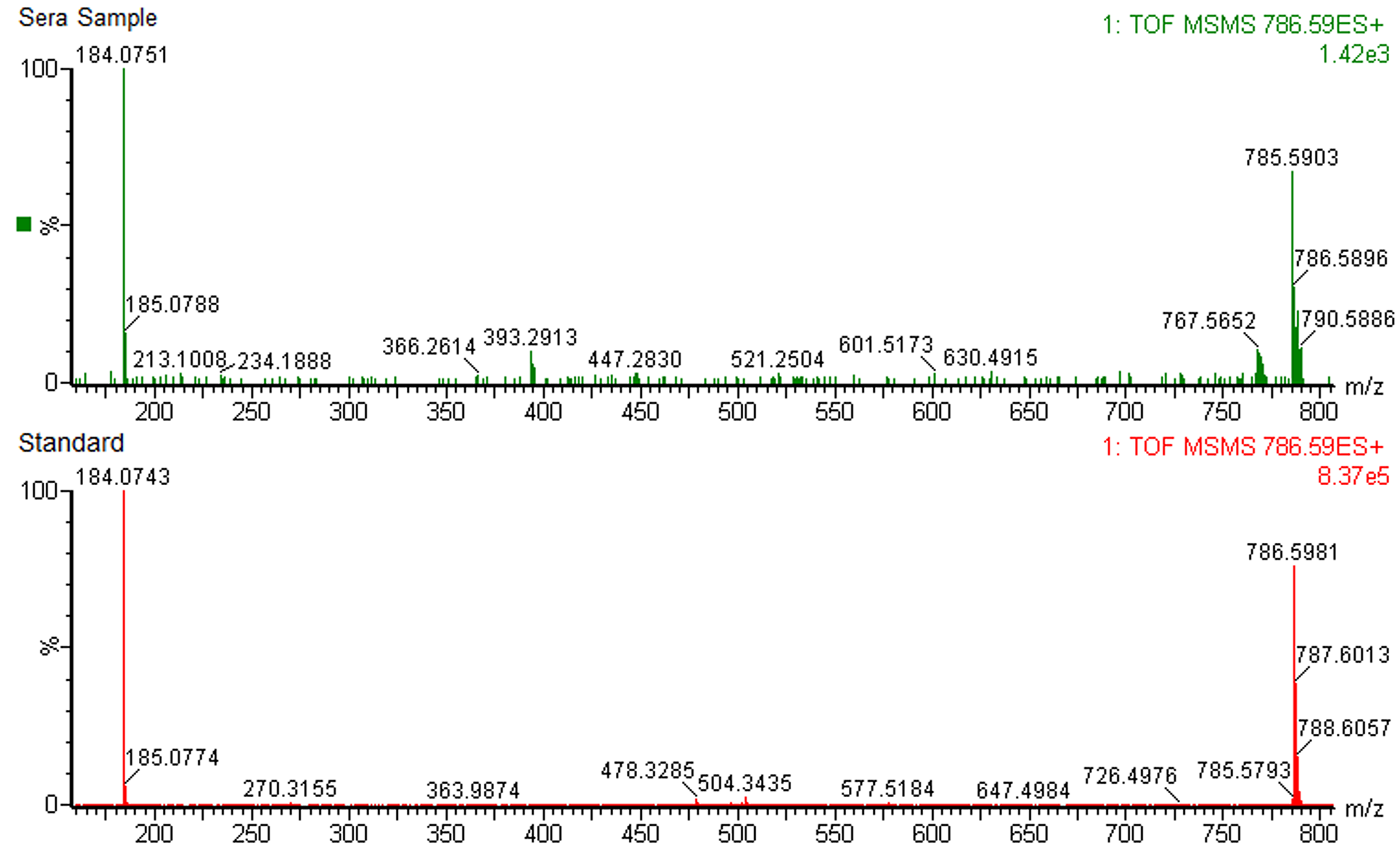

Supplement: Figure S2 — Validation of Phosphatidyl choline in the sample by MS/MS. Candidate marker was validated by UPLC-TOF-MS/MS. Q1 to Q3 transition of Phosphatidyl choline was compared with the ones in the sample (m/z 184.07, 185.07, 786.59, 787.60, 788.60). (TIF) [file pone.0102635.s002.tif]

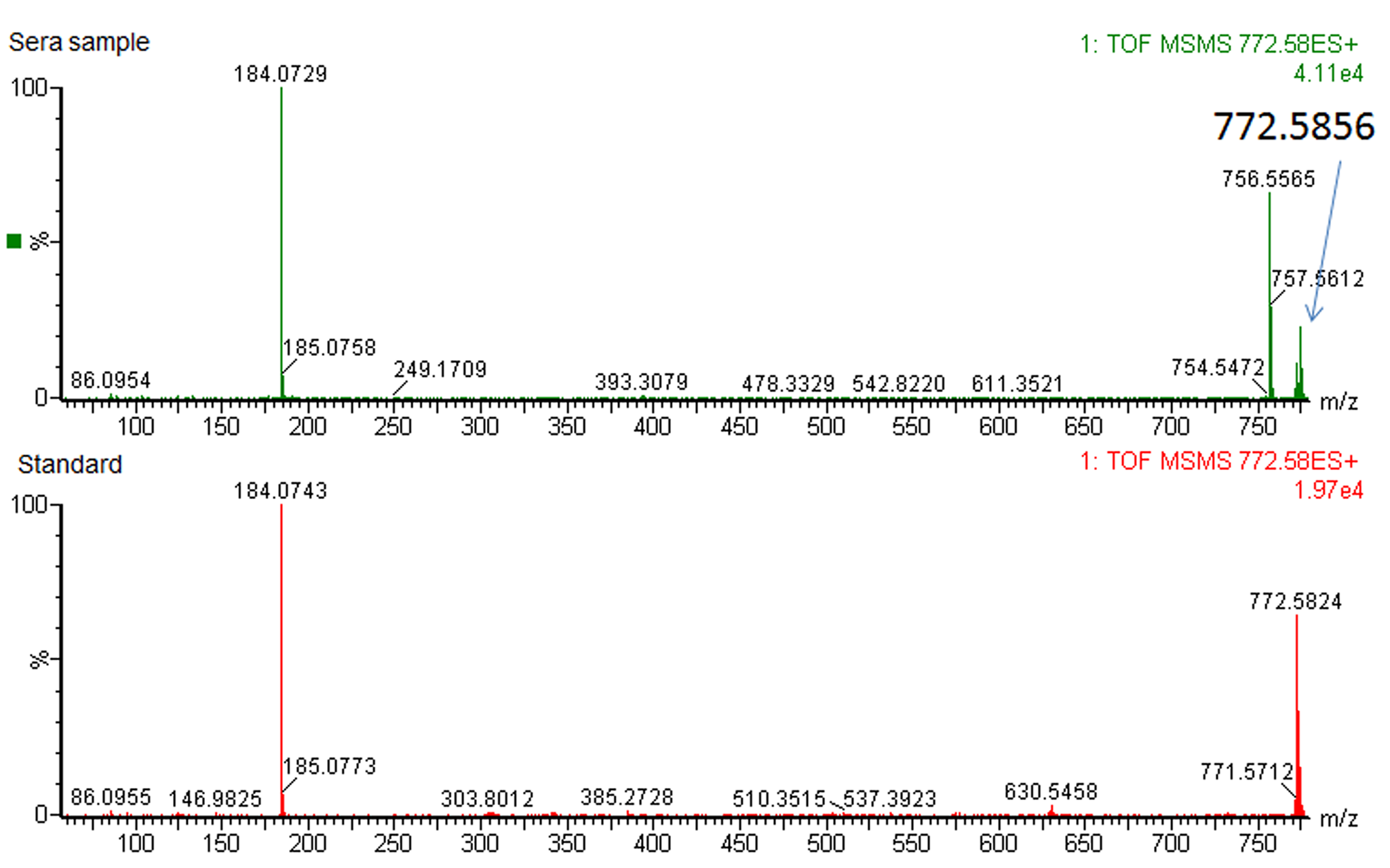

Supplement: Figure S3 — Validation of Phosphatidyl ethanolamine in the sample by MS/MS. Candidate marker was validated by UPLC-TOF-MS/MS. Q1 to Q3 transition of Phosphatidyl ethanolamine was compared with the ones in the sample (m/z 184.07, 185.07, 772.58). (TIF) [file pone.0102635.s003.tif]

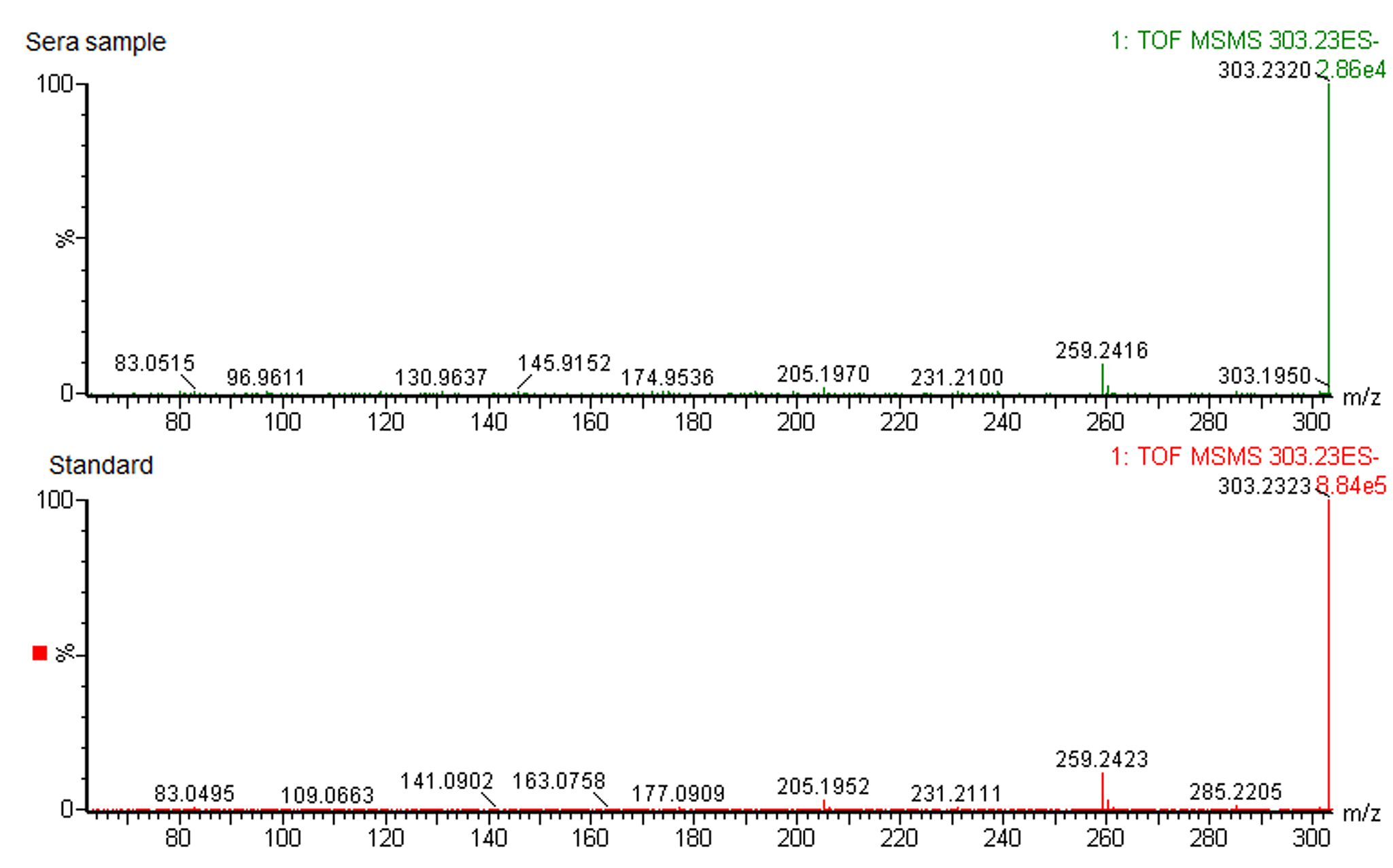

Supplement: Figure S4 — Validation of Arachidonic acid in the sample by MS/MS Validation of metabolites in the sample by MS/MS. Candidate marker was validated by UPLC-TOF-MS/MS. Q1 to Q3 transition of Arachidonic acid was compared with the ones in the sample (m/z 259.24, 303.23). (TIF) [file pone.0102635.s004.tif]
