# Supplementary material for: Serum Metabolomic Response of Myasthenia Gravis Patients to Chronic Prednisone Treatment
Source: PLoS One. 2014 Jul 17;9(7):e102635. doi: 10.1371/journal.pone.0102635 (PMC4102553; doi:10.1371/journal.pone.0102635)
Supplement: Table S1 — Random Forests top 50 metabolite putative identity lists in positive ionization mode. (DOCX) [file pone.0102635.s005.docx]

Table S1: RF top 50 metabolite putative identity list in positive ionization mode

| **No** | **m/z^1^** | **RT^2^ (min)** | **Fold Change^3^ T2/T1** | **p-value^4^** | **Metabolite^5^** | **ID^6^** |
| --- | --- | --- | --- | --- | --- | --- |
|  |  |  |  |  |  |  |
| 1 | 786.5977 | 9.11 | 9.96 ↑ | 7.4 E-09 | PC(18:1/18:1) | C00157 |
| 2 | 682.3608 | 6.18 | 9.71 ↑ | 4.1 E-07 | - | - |
| 3 | 790.6028 | 8.14 | 10.28 ↑ | 7.2 E-10 | PS(O-0:0/17:1) | - |
| 4 | 718.5643 | 8.77 | 8.83 ↑ | 5.5 E-11 | - | - |
| 5 | 654.3302 | 6.18 | 6.74 ↑ | 8.3 E-09 | A 80987 | C15661 |
| 6 | 606.5691 | 0.3 | 8.70 ↑ | 3.6 E-11 | - | - |
| 7 | 766.5699 | 8.5 | 7.62 ↑ | 6.9 E-06 | PC(P-18:1/18:3) | HMDB11312 |
| 8 | 801.5812 | 7.52 | 9.18 ↑ | 1.7 E-10 | - | - |
| 9 | 736.5399 | 9.09 | 16.71 ↑ | 1.4 E-07 | - | - |
| 10 | 787.6013 | 9.11 | 15.92 ↑ | 2.8 E-10 | GPCho(18:1/18:1) | HMDB05291 |
| 11 | 690.553 | 7.8 | 10.51 ↑ | 8.8 E-09 | - | - |
| 12 | 601.4644 | 5.12 | 6.27 ↑ | 7.2 E-08 | Hydroxy-spheroidenone | C15905 |
| 13 | 796.5731 | 8.59 | 15.53 ↑ | 4.8 E-10 | - | - |
| 14 | 718.8189 | 0.26 | 12.90 ↑ | 6.6 E-09 | - | - |
| 15 | 725.5515 | 8.12 | 6.87 ↑ | 4.4 E-12 | - | - |
| 16 | 807.5699 | 8.26 | 6.92 ↑ | 2.2 E-10 | GPCho(16:0/22:6) | HMDB05260 |
| 17 | 756.5522 | 8.16 | 9.22 ↑ | 7.5 E-10 | PC(16:0/18:3) | C00157 |
| 18 | 760.5822 | 9.01 | 11.53 ↑ | 2.2 E-07 | PC(20:0/14:1) | C00157 |
| 19 | 708.5069 | 9.11 | 8.27 ↑ | 3.0 E-07 | - | - |
| 20 | 776.5679 | 7.33 | 16.11 ↑ | 3.5 E-09 | - | - |
| 21 | 770.8434 | 0.26 | 8.75 ↑ | 4.8 E-07 | - | - |
| 22 | 761.3755 | 8.5 | 10.24 ↑ | 3.1 E-08 | - | - |
| 23 | 762.5873 | 9.01 | 10.02 ↑ | 6.6 E-08 | - | - |
| 24 | 817.5922 | 8.67 | 16.52 ↑ | 4.0 E-09 | PG(17:2/22:0) | - |
| 25 | 772.5844 | 8.79 | 12.94 ↑ | 7.2 E-09 | PE(20:1/18:1) | C00350 |
| 26 | 761.585 | 9.01 | 0.34 ↓ | 1.4 E-05 | - | - |
| 27 | 706.5423 | 8.36 | 0.45 ↓ | 7.9 E-05 | PE(12:0/21:0) | - |
| 28 | 828.8033 | 0.27 | 0.34 ↓ | 6.4 E-05 | - | - |
| 29 | 134.005 | 0.28 | 0.45 ↓ | 2.0 E-06 | - | - |
| 30 | 784.5812 | 8.58 | 0.01 ↓ | 1.4 E-03 | PC(17:2(9Z,12Z)/ 19:1(9Z)) | - |
| 31 | 750.6238 | 8.17 | 0.34 ↓ | 1.3 E-04 | - | - |
| 32 | 659.5124 | 8.34 | 0.38 ↓ | 2.6 E-03 | PE-Cer(d14:1/20:1) | - |
| 33 | 770.6033 | 8.88 | 0.39 ↓ | 9.3 E-05 | PC(P-18:1/18:1) | HMDB11275 |
| 34 | 728.5649 | 8.27 | 0.41 ↓ | 5.2 E-08 | PE(P-18:0/18:2) | HMDB11376 |
| 35 | 526.3752 | 5.44 | 0.46 ↓ | 4.7 E-07 | - | - |
| 36 | 600.4638 | 5.12 | 0.36 ↓ | 1.2 E-04 | - | - |
| 37 | 724.52 | 8.55 | 0.40 ↓ | 6.0 E-05 | - | - |
| 38 | 243.9141 | 9.39 | 0.45 ↓ | 4.7 E-04 | - | - |
| 39 | 593.5958 | 8.67 | 0.46 ↓ | 8.3 E-07 | - | - |
| 40 | 640.3194 | 6.18 | 0.39 ↓ | 1.9 E-04 | - | - |
| 41 | 808.5819 | 8.41 | 0.37 ↓ | 2.8 E-02 | PC(22:4/16:1) | C00157 |
| 42 | 617.5066 | 8.33 | 8.13 ↑ | 3.2 E-08 | - | - |
| 43 | 796.5441 | 7.3 | 5.17 ↑ | 3.7 E-08 | PS(P-18:0/20:4) | - |
| 44 | 751.5636 | 8.17 | 4.88 ↑ | 3.5 E-09 | - | - |
| 45 | 410.8589 | 0.28 | 2.58 ↑ | 1.3 E-08 | - | - |
| 46 | 832.5807 | 8.88 | 4.63 ↑ | 2.7 E-10 | PC(20:5/20:2) | C00157 |
| 47 | 837.6167 | 8.89 | 5.93 ↑ | 1.2 E-09 | GPCho(18:0/22:5) | HMDB05272 |
| 48 | 369.3524 | 8.33 | 0.49 ↓ | 1.9 E-05 | 3-Deoxyvitamin D3 | - |
| 49 | 135.0012 | 9.26 | 0.43 ↓ | 1.6 E-05 | - | - |
| 50 | 335.1713 | 8.5 | 0.43 ↓ | 9.1 E-05 | Veprisinium | C10745 |

^1^ mass/charge value. ^2^ retention time. ^3^Fold changes between post-treatment (T2) and pre-treatment (T1) groups. ^4^The p-value as determined by student’s t-test. ^5^Identified metabolites ^6^Metabolite ID obtained from Human Metabolome Data Base and KEGG.
