# Supplementary material for: Serum Metabolomic Response of Myasthenia Gravis Patients to Chronic Prednisone Treatment
Source: PLoS One. 2014 Jul 17;9(7):e102635. doi: 10.1371/journal.pone.0102635 (PMC4102553; doi:10.1371/journal.pone.0102635)
Supplement: Table S2 — Random Forests top 50 metabolite putative identity lists in negative ionization mode. (DOCX) [file pone.0102635.s006.docx]

Table S2 RF top 50 metabolite putative identity list in negative ionization mode

| **Query ID** | **m/z^1^** | **RT^2^ (min)** | **Fold Change3 T2/T1** | **p-value^4^** | **Metabolite^5^** | **ID^6^** |
| --- | --- | --- | --- | --- | --- | --- |
|  |  |  |  |  |  |  |
|  |  |  |  |  |  |  |
| 1 | 222.8419 | 8.01 | 0.37 ↓ | 1.4 E-06 | - | - |
| 2 | 200.8593 | 8.02 | 0.33 ↓ | 2.4 E-06 | - | - |
| 3 | 130.083 | 0.48 | 0.49 ↓ | 3.6 E-05 | - | - |
| 4 | 355.0169 | 2.15 | 0.78 ↓ | 7.6 E-09 | - | - |
| 5 | 369.2254 | 3.15 | 0.29 ↓ | 7.8 E-05 | TXB2 | C05963 |
| 6 | 257.8544 | 7.97 | 0.53 ↓ | 2.5 E-07 | - | - |
| 7 | 538.3145 | 4.45 | 16.62 ↑ | 2.4 E-10 | PS(19:0/0:0) | - |
| 8 | 632.3186 | 4.58 | 9.63 ↑ | 1.1 E-11 | - | - |
| 9 | 565.3344 | 4.58 | 12.01 ↑ | 2.2 E-12 | - | - |
| 10 | 440.6911 | 0.32 | 1.53 ↑ | 1.2 E-11 | - | - |
| 11 | 476.2722 | 4.56 | 6.60 ↑ | 2.8 E-10 | - | - |
| 12 | 590.3457 | 4.79 | 8.18 ↑ | 1.6 E-13 | - | - |
| 13 | 619.2883 | 4.46 | 5.07 ↑ | 1.6 E-10 | PI(20:4/0:0) | - |
| 14 | 614.3201 | 4.76 | 6.84 ↑ | 7.6 E-14 | - | - |
| 15 | 842.5567 | 7.54 | 19.45 ↑ | 1.3 E-12 | - | - |
| 16 | 674.5211 | 0.31 | 1.93 ↑ | 1.0 E-13 | - | - |
| 17 | 444.6776 | 0.32 | 1.61 ↑ | 3.1 E-11 | - | - |
| 18 | 827.5648 | 8.35 | 2.97 ↑ | 4.6 E-12 | - | - |
| 19 | 843.5597 | 7.54 | 16.73 ↑ | 3.2 E-12 | - | - |
| 20 | 589.3338 | 4.58 | 13.85 ↑ | 2.1 E-11 | - | - |
| 21 | 613.2727 | 4.78 | 5.03 ↑ | 1.4 E-13 | - | - |
| 22 | 612.271 | 4.78 | 5.34 ↑ | 6.7 E-13 | - | - |
| 23 | 566.3464 | 4.97 | 6.10 ↑ | 2.6 E-10 | PS(21:0/0:0) | - |
| 24 | 634.3343 | 4.98 | 6.06 ↑ | 6.6 E-11 | - | - |
| 25 | 564.3306 | 4.58 | 12.22 ↑ | 2.1 E-10 | - | - |
| 26 | 824.5455 | 8.08 | 6.83 ↑ | 2.1 E-12 | PS(17:2/22:2) | - |
| 27 | 500.6382 | 0.31 | 1.53 ↑ | 1.2 E-10 | - | - |
| 28 | 562.5907 | 0.31 | 1.61 ↑ | 1.1 E-11 | - | - |
| 29 | 504.3064 | 4.58 | 8.49 ↑ | 3.0 E-11 | PC(17:2/0:0) | - |
| 30 | 825.5486 | 8.08 | 6.72 ↑ | 9.1 E-12 | - | - |
| 31 | 609.3229 | 4.79 | 5.07 ↑ | 3.8 E-12 | - | - |
| 32 | 818.5567 | 7.33 | 16.13 ↑ | 3.7 E-11 | - | - |
| 33 | 826.5605 | 8.35 | 2.92 ↑ | 3.0 E-12 | PS(18:3/21:0) | - |
| 34 | 639.2857 | 4.97 | 4.30 ↑ | 1.4 E-09 | - | - |
| 35 | 564.5889 | 0.31 | 1.60 ↑ | 2.1 E-10 | - | - |
| 36 | 676.5128 | 0.31 | 1.90 ↑ | 4.5 E-12 | - | - |
| 37 | 678.5087 | 0.3 | 1.94 ↑ | 4.2 E-12 | - | - |
| 38 | 176.9953 | 0.35 | 1.92 ↑ | 1.8 E-11 | - | - |
| 39 | 543.3387 | 4.8 | 5.07 ↑ | 5.4 E-11 | - | - |
| 40 | 588.3307 | 4.58 | 15.22 ↑ | 1.5 E-11 | - | - |
| 41 | 794.4262 | 0.3 | 1.79 ↑ | 1.5 E-10 | - | - |
| 42 | 502.6347 | 0.32 | 1.52 ↑ | 1.0 E-11 | - | - |
| 43 | 798.418 | 0.3 | 1.83 ↑ | 8.9 E-11 | - | - |
| 44 | 530.3012 | 4.8 | 4.95 ↑ | 1.4 E-12 | - | - |
| 45 | 820.564 | 7.33 | 11.15 ↑ | 2.4 E-11 | - | - |
| 46 | 792.5368 | 8.54 | 1.75 ↑ | 3.5 E-10 | - | - |
| 47 | 92.929 | 0.33 | 1.24 ↑ | 2.1 E-08 | - | - |
| 48 | 685.2535 | 4.79 | 5.29 ↑ | 1.6 E-12 | - | - |
| 49 | 819.5599 | 7.33 | 15.77 ↑ | 5.7 E-11 | - | - |
| 50 | 506.3229 | 4.97 | 5.68 ↑ | 3.5 E-10 | LysoPE(20:1/0:0) | HMDB11512 |

^1^ mass/charge value. ^2^ retention time. ^3^Fold changes between post-treatment (T2) and pre-treatment (T1) groups. ^4^The p-value as determined by student’s t-test. ^5^Identified metabolites ^6^Metabolite ID obtained from Human Metabolome Data Base and KEGG.
